# Crosstalk between β-carbonic anhydrases and PsbS in the regulation of photosynthesis and stress tolerance in Arabidopsis

**DOI:** 10.1093/jxb/erag155

**Published:** 2026-04-02

**Authors:** Alex Białas, Joanna Dąbrowska-Bronk, Piotr Gawroński, Stanisław Karpiński

**Affiliations:** Department of Plant Genetics, Breeding and Biotechnology, Institute of Biology, Warsaw University of Life Sciences, Warsaw 02-776, Poland; Department of Biochemistry and Molecular Biology, Centre of Translational Research, Centre of Postgraduate Medical Education, Warsaw, Poland; Department of Botany and Plant Physiology, Institute of Biology, Warsaw University of Life Sciences, Warsaw 02-776, Poland; Department of Plant Genetics, Breeding and Biotechnology, Institute of Biology, Warsaw University of Life Sciences, Warsaw 02-776, Poland; Department of Plant Genetics, Breeding and Biotechnology, Institute of Biology, Warsaw University of Life Sciences, Warsaw 02-776, Poland; University of Glasgow, UK

**Keywords:** Bicarbonate, carbonic anhydrases, CO_2_ assimilation, pH homeostasis, photooxidative stress, photoprotection, photosynthesis, PsbS, water use efficiency

## Abstract

Plant growth and stress responses are tightly linked to chloroplast retrograde signaling. Key regulators, such as the 22 kDa PSII protein (PsbS) and β-carbonic anhydrases (βCAs), have been implicated in photoprotection and stress acclimation. In this study, we investigated the effects of simultaneously overexpressing *βCA1* and/or *βCA2* in a PsbS-overexpressing *Arabidopsis thaliana* background. Double and triple transgenic lines (oePsbSoeβCA1 and oePsbSoeβCA1βCA2) showed enhanced photoprotection, improved acclimation to fluctuating light, and greater water use efficiency, but at the cost of reduced biomass relative to Col-0 and the *npq4-1* mutant. Following bicarbonate fertilization, the triple overexpression line had improved biomass compared with oePsbS and *npq4-1*, but not compared with Col-0. Importantly, our data reveal that βCAs modulate PsbS abundance, supporting the existence of their crosstalk. Bicarbonate treatment activated stress-responsive genes and transcription factors exclusively in oePsbSoeβCA lines, indicating heightened sensitivity associated with elevated activity of βCAs. Together, these findings suggest a previously unrecognized regulatory link between the activity of βCAs and PsbS turnover in fine-tuning stress responses and productivity, mediated at least in part by changes in PsbS expression. However, the underlying molecular mechanisms require further investigation to determine whether these effects are specific to the PsbS level or reflect a broader role for βCAs.

## Teaser

In Arabidopsis, βCA1 and βCA2 modulate PsbS abundance and stress responses via chloroplast pH and bicarbonate metabolism, directly influencing photosynthetic energy balance and non-photochemical quenching mechanisms.

## Introduction

Oxygenic photosynthesis is the foundation of life on Earth, enabling plants to convert light energy, carbon dioxide (CO_2_), and water (H_2_O) into organic compounds and oxygen (O_2_) (Stirbet *et al.*, 2020). To balance the absorption of light energy with the demands of photochemistry, plants have evolved sophisticated photoprotective mechanisms, most notably non-photochemical quenching (NPQ), which safely dissipates excess excitation energy as heat and fluorescence (Müller *et al.*, 2001). NPQ consists of a ΔpH and PsbS-dependent process (qE), photoinhibition (qI), state transition (qT), and zeaxanthin formation (qZ) (Baker, 2008). The 22 kDa PSII subunit PsbS protein, belonging to the Chl *a*/*b*/xanthophyll-binding proteins [light-harvesting complex (LHC)] superfamily, is a central component of the NPQ response, acting as a sensor of lumenal pH and facilitating rapid photoprotection under fluctuating and high-light conditions (Takizawa *et al.*, 2007; Johnson and Ruban, 2010; Fan *et al*., 2015). Recently, it was demonstrated that the PsbS protein level, as well as local, systemic, and network wave-like changes in NPQ, influences the plastoquinone (PQ) pool redox status, and thus electrical, ΔpH-, abscisic acid (ABA)-, salicylic acid (SA)-, and jasmonic acid (JA)-dependent retrograde signaling pathways from chloroplasts. PsbS regulation and its involvement in chloroplast retrograde signaling—whereby chloroplast status influences nuclear gene expression—has emerged as a key determinant of plant acclimation, stress tolerance, and productivity (Mühlenbock *et al.*, 2008; Szechyńska-Hebda *et al.*, 2010, 2022; Ciszak *et al*., 2015; Gururani *et al.*, 2015; Górecka *et al.*, 2020; De Ollas *et al.*, 2021; De Souza *et al.*, 2022; Molinari *et al.*, 2023; Kamran *et al*., 2025, 2026; Zarrin Ghalami *et al*., 2026).

Nowadays, many researchers wonder how to improve photosynthesis for C_3_ and C_4_ plants, and thus biomass and seed yield during the time of global warming. There are some suggested transgenic modifications to improve photosynthesis of C_3_ plants, such as introducing the CO_2_-concentrating mechanism from algae or C_4_ plants, optimizing the electron transport chain (ETC) and photorespiration, improving recovery of NPQ and light absorption/conversion, and modifying Calvin cycle enzymes (Price *et al.*, 2013; Ort *et al.*, 2015; Kromdijk *et al.*, 2016). So far, to improve photoprotective mechanisms and biomass production, overexpression of genes coding for violaxanthin deepoxidase (VDE), PsbS, and zeaxanthin epoxidase (ZEP) has been provided in different plant species. Overexpression of *VDE*, *PsbS*, and *ZEP* in tobacco and soybean plants showed improved NPQ, seed yield, and biomass production (Kromdijk *et al.*, 2016; De Souza *et al*., 2022). Conversely, in Arabidopsis and potato plants, overexpression of the same genes resulted in improved NPQ but reduced growth under fluctuating light (Garcia-Molina and Leister, 2020; Lehretz *et al.*, 2022). *Arabidopsis thaliana* overexpressed PsbS protein had a higher qE capacity during short-term high-light conditions. Transgenic plants were significantly more tolerant to transient photoinhibition expressed by having higher PSII maximal photochemical efficiency (*F*_v_/*F*_m_), in contrast to the *npq4-1* mutant, which lacks functional PsbS (Li *et al.*, 2002; Górecka *et al.*, 2020). Arabidopsis plants lacking the functional PsbS gene and protein (*npq4-1* mutant) after excess light stress could not establish an optimal fluorescence decay time in comparison with wild-type plants with a functional PsbS gene and protein (Ciszak *et al*., 2015). These results indicate that increased photosynthetic productivity by, for example, improved relaxation of photoprotection, not only depends on the species and its morphology, but is strictly related to the physiological and molecular basis of manipulated processes.

Recent studies have expanded knowledge about proteins involved in chloroplast signaling, including β-class carbonic anhydrases (βCAs). βCAs are the most abundant in land plants, catalyzing the reversible hydration of CO_2_ to bicarbonate (HCO_3_^−^), supporting CO_2_ supply for photosynthesis and influencing pH homeostasis (Moroney *et al*., 2001). In C_3_ plants, βCAs are localized to multiple subcellular compartments, with βCA1 targeted to the chloroplast and βCA2 predominantly to the cytosol, although the subcellular localization of CAs and their isoforms varies among species. Beyond their well-defined function, βCAs have been implicated in stomatal regulation, amino acid and lipid biosynthesis, and responses to both biotic and abiotic stresses (DiMario *et al.*, 2017; Kolbe *et al.*, 2018). Notably, βCA activity has been linked to the regulation of NPQ and the photosynthetic ETC, suggesting a potential interplay with photoprotective and signaling pathways (Dąbrowska-Bronk *et al.*, 2016; Shitov *et al.*, 2018).

Indeed, CAs participate in plant acclimation to diverse stresses, including drought, high light, elevated CO_2_, heat, salinity, inorganic carbon deficit, excess HCO_3_^−^, and pathogen attack (Wang *et al.*, 2016; Kolbe *et al.*, 2018; Hu *et al.*, 2021; Polishchuk, 2021). Their expression patterns vary depending on stress type, intensity, and plant species, and our previous findings suggested that CAs may contribute to HCO_3_^−^ detoxification and facilitate its utilization in photosynthesis (Dąbrowska-Bronk *et al.*, 2016). Furthermore, recent work has shown that, under stress conditions, chloroplast βCAs and their isoforms can be translocated into the cytoplasm and nucleus, where they interact with proteins of the SA signaling cascade, although the physiological significance of this phenomenon remains unclear (Medina-Puche *et al.*, 2017).

Given the emerging roles of βCAs and PsbS in photosynthetic and photoprotective mechanisms in C_3_ plants, we tested the combined effect of *βCA1* and/or *βCA2* overexpression in the *A. thaliana* PsbS-overexpressing background. We aimed to determine how this genetic combination influences photosynthetic parameters, biomass production, and stress responses, particularly under bicarbonate fertilization. The results demonstrated that overexpression of *βCA1* and/or *βCA2* enhanced photoprotection and water use efficiency (WUE) but reduced stomatal conductance (*g*_s_) and transpiration rate, leading to decreased biomass yield. Recent studies have identified βCAs and PsbS as important regulators of plant stress responses, beyond their well-known function in carbon metabolism and photoprotection, respectively. We also observed that overexpression of *βCA1* and/or *βCA2* in a PsbS-overexpressing background leads to a substantial decrease in PsbS protein level, suggesting a previously unrecognized regulatory interaction. Together, these findings provide new insights into the complex interplay between CAs, PsbS, and inorganic carbon in plant acclimation, while highlighting key limitations and the need for future research.

## Materials and methods

### Plant material and growth conditions

#### General growth conditions

Wild-type, mutant, and transgenic plants were germinated on Jiffy pots (Jiffy Products, The Netherlands). Pots were kept for 48 h at 4 °C and then placed in laboratory conditions—long-day photoperiod (16 h light/8 h dark) in 120 µmol m^–2^ s^–1^ [normal light (NL)], 80 µmol m^–2^ s^–1^ [low light (LL)], and 800 µmol m^–2^ s^–1^ [high light (HL)], and a temperature range of 20/22 °C in a growth chamber (BDR16, Conviron, UK). All experiments were conducted on *A. thaliana* plants of ecotype Columbia 0 (Col-0), *βca1* and *βca2* mutants (Dąbrowska-Bronk *et al*., 2016), the *npq4-1-1* mutant—devoid of the the *PsbS* gene (AT1G44575)—the transgenic line overexpressing PsbS protein (oePsbS) (Li *et al.*, 2002), and transgenic lines overexpressing combined/single *βCA1* (AT3G01500) and *βCA2* (AT5G14740).

#### Bicarbonate fertilization

Wild-type, mutant, and transgenic plants were grown in normal light conditions (120 µmol m^–2^ s^–1^) with a long photoperiod (16/8 h light/dark) in the growing chamber. Growth temperature was 21 °C and humidity was 50–60%. Two-week old plants were fertilized every 2 d with 10 ml of 3 mM NaHCO_3_ (pH 7, buffered with 5 M KOH; maintaining HCO_3_^−^ as the dominant form in solution). Control plants were treated with water at the same time and with the same volume.

#### Fresh and dry weight measurements

FW was collected from 4-week-old plants (±1 mg). Then the rosettes were dried for 24 h at 150 °C in a Hereus® drier and weighed to establish DW (±0.1 mg).

### Transgenic plants generation

*βCA1* (1044 bp) and *βCA2* (996 bp) coding sequences (The Arabidopsis Information Resource; www.arabidopsis.org) were amplified based on Arabidopsis Col-0 ecotype cDNA using Phusion II hot start high fidelity polymerase (Thermo Fisher Scientific, UK) and cloned into the pJET 1.2 vector according to the manufacturer’s instructions (Thermo Fisher Scientific, UK). Overexpression constructs for *βCA1* and *βCA1βCA2* were generated using binary vectors pK7FWG2, driven by the 35S promoter, and pK7m34GW2-8m21GW3D, driven by the *ROLD* and *35S* promoters, respectively, using ‘Gateway’ technology (Karimi *et al.*, 2002), according to the manufacturer’s instructions (Thermo Fisher Scientific, UK). Arabidopsis Col-0 and single PsbS-overexpressing plants were transformed with pK7FWG2::*βCA1* and pK7m34GW2-8m21GW3D::*βCA1βCA2* constructs based on *Agrobacterium*-mediated stable transformation using the floral-dip method (Clough and Bent, 1998). Finally, eight and 10 independent transgenic lines for Col-0::oeβCA1 and Col-0::oeβCA1βCA2, and seven and six independent transgenic lines for oePsbS::oeβCA1 and oePsbS::oeβCA1βCA2 were obtained, respectively (T_3_ generation homozygous plants). Three of them, with the highest overexpression of *βCA1* and *βCA1βCA2* genes, were chosen for further analysis.

### Gas exchange measurements

Gas exchange parameters such as net CO_2_ assimilation rate, transpiration, *g*_s_, and WUE were measured using the CIRAS-3 Portable Photosynthesis System according to the manufacturer’s instructions (PP Systems, Amesbury, MA, USA). Eight leaves were collected per genotype. Measurements were performed in a constant light intensity of 120 µmol m^–2^ s^–1^. The temperature, CO_2_ concentration, and humidity in the measuring chamber were maintained at 25 °C, 400 ppm, and 60%, respectively.

### 3-(3,4-Dichlorophenyl)-1,1-dimethylurea and high-light treatments

Leaf disks cut from 4-week-old plants were treated with 25 μM 3-(3,4-dichlorophenyl)-1,1-dimethylurea (DCMU) and with 1000 µmol m^–2^ s^–1^ of white light for 30 min, then were kept in the growing chamber under ambient light between measurements. The maximum quantum yield of PSII (QY_max_) of leaf disks and rosettes was measured using FluorCam 800MF (Photon Systems Instruments, Czech Republic). As a control, we used leaf disks incubated in identical conditions without herbicide and plants non-treated with high light, respectively.

### Chlorophyll *a* fluorescence

Chl *a* fluorescence was measured using an imaging chlorophyll fluorometer (FluorCam 800 MF, PSI, Czech Republic). Prior to measurements, the plants were dark adapted for 30 min to determine *F*_0_ and *F*_m_. Fluorescence parameters were determined according to the manufacturer’s instructions and previously described procedures (Baker, 2008). At least six plants were measured per genotype.

### Analysis of quantum efficiency of PSI and PSII

Light–response curves of the complementary quantum yields of PSI and PSII were measured at absorbance changes—830 nm and 875 nm—with a Dual-PAM-100 (Walz GmbH, Germany). At least four plants were measured per genotype. Analysis was performed on the fifth to seventh detached leaves, which were dark-adapted for 30 min. The parameters of PSI and PSII activity were calculated according to the manufacturer’s instructions and Niewiadomska *et al.* (2011).

### Thylakoid delta pH measurements

Electrochromic shift (ECS) spectroscopic measurements were conducted using the DualPAM P515 module (Walz, Germany), which allows simultaneous measurements of the dual-beam 550–515 nm difference signal (Schreiber and Klughammer, 2008). Prior to measurements, plants were dark-adapted for 30 min before the 200 µmol m^–2^ s^–1^ actinic light (AL) was switched on. After 15 min of AL illumination, the ECS signal was measured for 2 min, and the dark relaxation of the ECS signal was measured for 50 s. Immediately after the first measurement, the ECS signal was again recorded for 2 min after exposure to 660 µmol m^–2^ s^–1^ AL, and for 50 s after switching it off. The protocol for ECS measurements of the proton motive force (pmf) components (ΔΨ and ΔpH) was based on a previously described method (Bailleul *et al.*, 2010). Proton permeability was evaluated as gH^+^=1/τ after fitting the decay kinetics of the ECS signal in the first 100 ms of dark relaxation with a single exponential. At least four plants were measured per genotype. Analysis was performed on the fifth to seventh detached leaves after dark adaptation for 30 min.

### RNA isolation, sequencing, and real-time reverse transcription–PCR analysis

Total RNA was extracted using the Spectrum™ Plant Total RNA kit (Sigma Aldrich, Germany) according to the manufacturer’s recommendations. Prior to quantitative PCR (qPCR) analysis, RNA samples were subjected to DNase I treatment with the DNAfree™ kit (Ambion, Applied Biosystems, UK) for 30 min. Total RNA concentration was determined using a UV–VIS spectrophotometer (Thermo Fisher Scientific, NanoDrop™, UK). Reverse transcription reaction for cDNA synthesis was performed on 2 μg of RNA using the High Capacity cDNA Reverse Transcription Kit (Applied Biosystems, UK) according to the manufacturer’s instructions. Real-time qPCR was performed with 2.5 ng ml^–1^ cDNA using the 7500 Fast Real-Time PCR System (Applied Biosystems, UK) according to the manufacturer’s instructions. Relative transcript levels were determined using 7500 Software (Applied Biosystems, UK) by normalizing the threshold cycle number of each gene with the *A. thaliana* protein phosphatase 2A (*PP2AA3*; AT1G13320) reference gene. Relative gene expression was calculated using the 2^−ΔΔCT^ method (Rao *et al.*, 2013). Primer sequences used in this work are available in Supplementary Table S1. According to transcriptome analysis, RNA quality was tested by using the Expirion™ Automated Electrophoresis System (Bio-Rad, Germany). Further, samples were processed at Macrogen (Seoul, Korea). Libraries were constructed using a TruSeq Stranded mRNA Sample Prep Kit (Illumina, San Diego, CA, USA), and sequencing was conducted on the NovaSeq platform in 2×150 bp mode. The Arabidopsis genome sequence, annotation, and annotated sequence features were downloaded from TAIR Ensembl (Ensembl Plants, version 52 TAIR10 genome release). Reads were mapped to *A. thaliana* cDNAs (Ensembl, TAIR 10, release 41) using Salmon software (Patro *et al.*, 2017). Transcript-level abundances were imported into R and analyzed using the DESeq2 package (Love *et al.*, 2014). Genes with log2 FC (fold change) value >1 or < −1 and with adjusted *P*-value <0.05 were considered as significantly affected. Gene Ontology (GO) enrichment analysis of gene sets was conducted using the topGO package from Bioconductor. Each qPCR and transcriptomic analyses were performed for three biological samples and three technical repeats.

### Protein extraction and western blotting

Total protein was isolated with 4× Laemmli Buffer (Bio-Rad, Germany) from plant leaf tissue ground in liquid N_2_. Double and triple transgenic lines are presented as a pooled material of three independent transgenic lines for each construct. Protein concentration in samples was quantified using the RC DC Protein Assay (Bio-Rad, Germany). Samples were separated by SDS–PAGE on a 10% acrylamide gel; each well was loaded with 20 μg of total protein. After separation, the samples were transferred from the gel to an Immobilon P polyvinylidene fluoride (PVDF) membrane (Merck, Germany) by semi-dry transfer, then blocked with 1% non-fat dry milk (Bio-Rad) overnight at 4 °C. The dilutions of primary and secondary antibodies are presented in Supplementary Table S2. Chemiluminescence signal was detected using the Pierce ECL Plus Western Blotting Substrate (Thermo Fisher Scientific, UK) and ChemiDoc Imaging Systems (Bio-Rad, Germany). To confirm that wells were loaded with the same amount of protein, analogous gels were stained with QC Colloidal Coomassie Stain (Bio-Rad, Germany).

### Photosynthetic pigment analysis

Four-week old plants were ground in liquid N_2_, then ∼10–15 mg of tissue was suspended in pure methanol. The analysis of Chl *a*, Chl *b*, and carotenoid contents was performed using Multiskan GO (Thermo Fisher Scientific, UK) as described before by Gawroński *et al.* (2021). Absorbance was measured at three wavelengths: 663, 645, and 470 nm. Calculations were performed as reported previously by Sumanta *et al.* (2014).

### Statistical analyses

Statistics for all the results are presented as the means ±SDs. The significant differences were revealed by Fisher’s least significant difference (LSD) procedure with 95% confidence intervals. Statistical analyses were performed in STATGRAPHICS XVII-X64 software.

## Results

### Overexpression of *βCA1* and *βCA2* decreased the PsbS level

As described in the Materials and methods, seven and six independent transgenic lines were generated for the single (oePsbS::oeβCA1) and double (oePsbS::oeβCA1βCA2) constructs in the *A. thaliana* PsbS-overexpressing background, respectively. Three independent lines with the highest transgene expression were selected for further analysis. Relative transcript levels and immunoblotting results are presented in Supplementary Fig. S1. *Arabidopsis thaliana* Col-0, the *npq4-1* mutant, and oePsbS were used as reference genotypes. Immunoblot analysis revealed that in both double and triple oePsbSβCA lines, PsbS protein levels were markedly reduced compared with oePsbS, approaching wild-type (Col-0) levels (Supplementary Fig. S1C, D). To validate these findings, PsbS transcript and protein abundance were also analyzed in Arabidopsis Col-0::oeβCA1 and Col-0::oeβCA1βCA2 lines (Supplementary Fig. S2A). Both analyses showed a reduction in PsbS expression and protein content relative to Col-0. Conversely, PsbS expression was elevated in Arabidopsis *βca* knockout mutants, supporting a regulatory relationship between the activity of βCAs and PsbS abundance (Supplementary Fig. S2B).

### Overexpression of *βCA1* and *βCA2* in the oePsbS background deregulates photosynthesis and biomass production

**Fig. 1.**
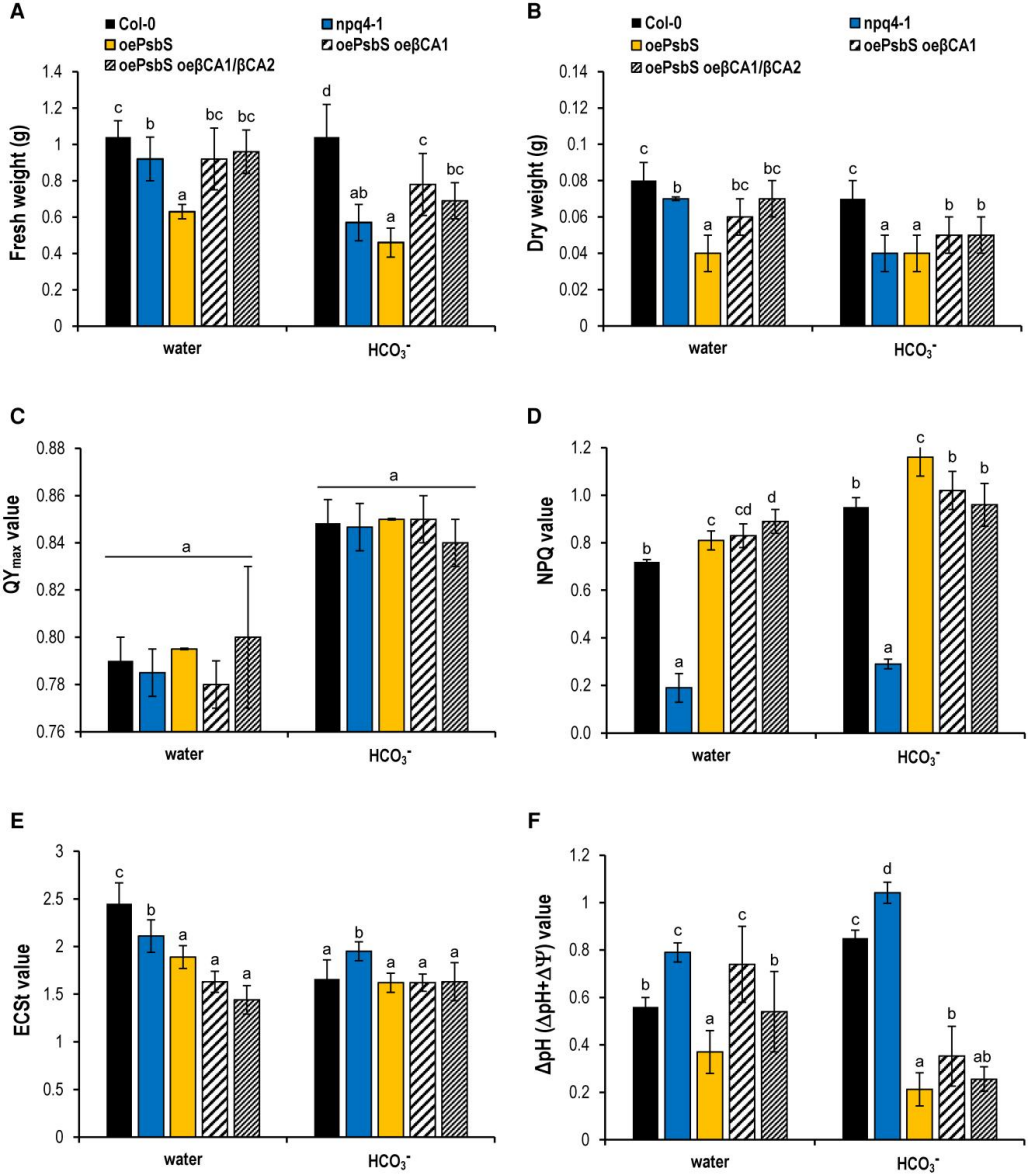
Overexpression of βCA1 and βCA2 influenced biomass production, NPQ, and ΔpH after water and bicarbonate supply. Effects on biomass productivity and photosynthetic parameters of bicarbonate fertilization of Col-0, *npq4-1*, oePsbS, double (oePsbSoeβCA1), and triple (oePsbSoeβCA1βCA2) overexpressing transgenic *A. thaliana* lines cultivated in ambient laboratory conditions. (A) FW and (B) DW of 4-week-old plants. (C) Maximum yield of PSII (QY_max_) and (D) non-photochemical quenching (NPQ). (E) Total proton motive force and (F) ΔpH parameters. Plants were cultivated in a growing chamber under normal light conditions of 120 µmol m^–2^ s^–1^, fertilized with water or 3 mM HCO_3_^–^. One-way ANOVA Fisher’s least significant difference (LSD) procedure, 95% confidence interval, was used to estimate the difference between each pair of means. Data are shown as means ±SD (*n*=8 A–D, *n*=6 E, F). Results for double and triple transgenic lines are presented as an average value of three independent transgenic lines for each construct.

To confirm the effect of *βCA1* and *βCA2* overexpression on photosynthetic efficiency in oePsbS::oeβCA1 and oePsbS::oeβCA1βCA2 lines, we measured FW and DW, NPQ, QY_max_, total pmf, and the thylakoid ΔpH, in 4-week-old plants following water or HCO_3_^−^ treatment (Fig. 1). Under laboratory conditions, single PsbS-overexpressing lines exhibited higher NPQ values and a stabilized pH balance between the chloroplast lumen and stroma (reduced ΔpH) after bicarbonate fertilization, as expected. In contrast, both double and triple oePsbSoeβCA lines displayed a partial reversion of these phenotypes: NPQ and ΔpH values were intermediate between those of oePsbS and Col-0, closely corresponding to the reduced PsbS protein abundance in these lines (Fig. 1D, F; Supplementary Fig. S1). No significant differences in QY_max_ were observed, indicating that the growth conditions did not impair PSII photochemistry (Fig. 1C).

These trends in NPQ and ΔpH were mirrored in biomass production after bicarbonate fertilization. While single PsbS-overexpressing plants maintained comparable biomass accumulation regardless of fertilization, oePsbSoeβCA lines showed a significant increase in biomass, probably linked to the partial reduction of PsbS protein levels in these lines (Fig. 1A, B). Gas exchange measurements further revealed that double and triple oePsbSoeβCA lines exhibited reduced *g*_s_ and transpiration, resulting in enhanced WUE compared with Col-0 and oePsbS (Supplementary Fig. S3).

Due to the fact that CO_2_ assimilation is driven by the light reaction products of photosynthesis—ATP and NADP—determination of the influence *βCA1* and *βCA2* overexpression in a PsbS-overexpressing background on photosynthetic productivity was also carried out in the greenhouse, under variable natural light conditions with bicarbonate fertilization (Supplementary Fig. S4). Triple oePsbSoeβCA1βCA2 lines exhibited improved biomass accumulation compared with oePsbS, suggesting either an additive effect of βCAs or a pivotal role for βCA2 in HCO_3_^−^/CO_2_ transport. Moreover, these findings point to a potential role for βCAs in fine-tuning regulatory responses to fluctuating environmental conditions such as excess light and high-temperature stress. Despite growing conditions, double and triple *βCA* overexpression lines did not exhibit greater biomass accumulation than Col-0 plants.

### Overexpression of *βCA1* and *βCA2* in the oePsbS background improved photoinhibition and PSII photoprotection

**Fig. 2.**
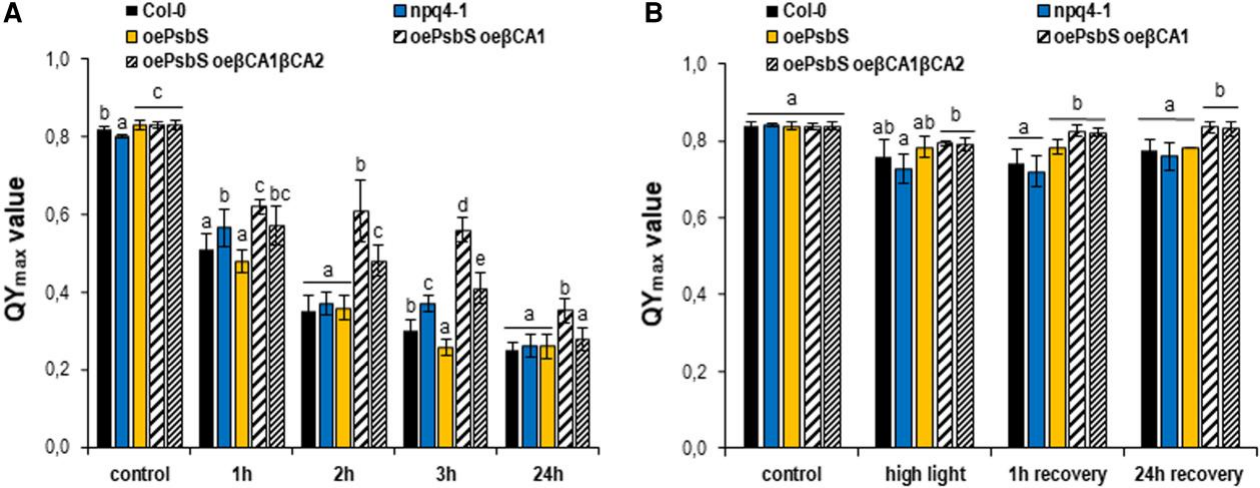
Overexpression of βCA1 and βCA2 led to improved photoprotection during and after photoinhibition. Effects on maximal photochemical efficiency of PSII (QY_max_) after DCMU and HL treatments of Col-0, *npq4-1*, oePsbS, double (oePsbSoeβCA1), and triple (oePsbSoeβCA1βCA2) overexpressing transgenic *A. thaliana* lines cultivated in ambient laboratory conditions. (A) QY_max_ after 25 µM DCMU treatment and (B) HL (1000 µmol m^–2^ s^–1^) treatment. Four-week-old plants were cultivated in a growing chamber under laboratory ambient light conditions (120 µmol m^–2^ s^–1^) and exposed for 30 min to HL. One-way ANOVA Fisher’s least significant difference (LSD) procedure, 95% confidence interval, was used to estimate the differences between each pair of means. Data are shown as means ±SD (*n*=8). Results for double and triple transgenic lines are presented as an average value of three independent transgenic lines for each construct.

**Fig. 3.**
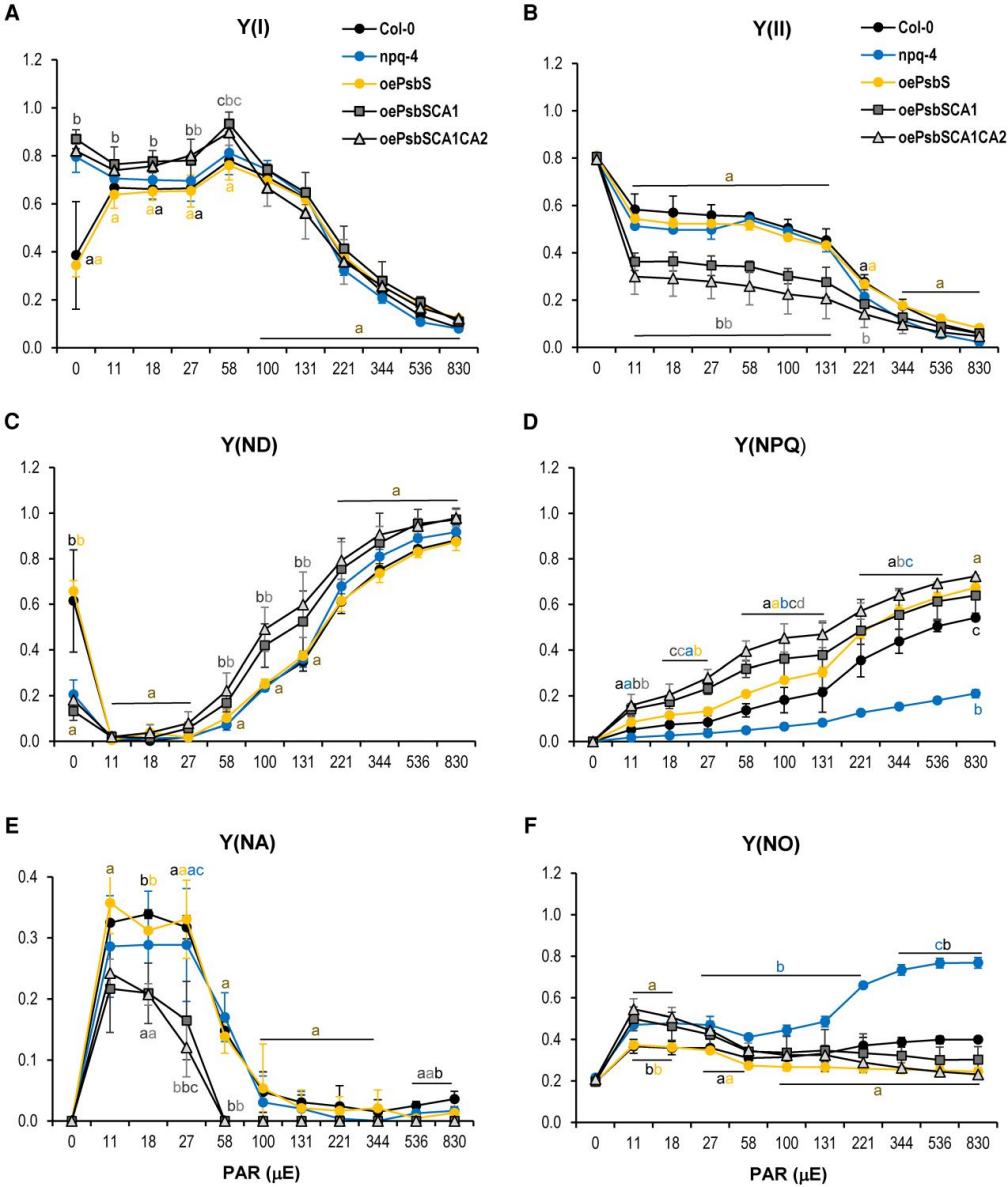
Overexpression of βCA1 and βCA2 influenced energy distribution and dissipation between photosystems. Light–response curves of the complementary quantum yields of PSI and PSII of Col-0, *npq4-1*, oePsbS, double (oePsbSoeβCA1), and triple (oePsbSoeβCAβCA2) overexpressing transgenic *A. thaliana* lines cultivated in ambient laboratory conditions (A–F). Four-week-old plants were cultivated in a growing chamber under normal light conditions of 120 µmol m^–2^ s^–1^ Complementary quantum yields were measured at each PAR value (0–830 µmol m^–2^ s^–1^). One-way ANOVA Fisher’s least significant difference (LSD) procedure, 95% confidence interval, was used to estimate the difference between each pair of means. Data are shown as means ±SD (*n*=8). Results for double and triple transgenic lines are presented as an average value of three independent transgenic lines for each construct. Y(I), quantum yield of photochemical energy conversion in PSI; Y(ND), quantum yield of non-photochemical energy dissipation due to donor-side limitation in PSI; Y(NA), quantum yield of non-photochemical energy dissipation due to acceptor-side limitation in PSI; Y(II), quantum yield of photochemical energy conversion in PSII; Y(NPQ), non-photochemical quenching; Y(NO), non-regulated energy dissipation.

QY_max_ and NPQ are sensitive indicators of PSII performance, reflecting changes that can lead to photoinhibition, D1 protein degradation, photooxidative stress, and ultimately the induction of cell death (Mateo *et al.*, 2004). HL and DCMU are commonly used to inhibit PSII function. DCMU blocks electron transfer from Q_A_ to the PQ pool, slowing Q_A_ oxidation and limiting PQ reduction. Treatment with DCMU strongly suppresses NPQ, induces cyclic electron transport (CET), and inhibits excess-light-mediated stomatal closure as well as PQ redox-dependent retrograde signaling involved in light acclimation and immune responses (Mühlenbock *et al.*, 2008; Szechyńska-Hebda *et al.*, 2010). In our experiments, plants were treated with DCMU and exposed to HL to induce photoinhibition. Double and triple oePsbSoeβCA lines maintained significantly higher PSII QY_max_ than all other genotypes following both DCMU (Fig. 2A) and HL treatment (Fig. 2B). These findings indicate that overexpression of *βCA1* and *βCA2* confers enhanced tolerance to moderate photoinhibition and HL stress, probably regardless of PsbS protein abundance. Since both DCMU and HL stimulate CET around PSI, we next analyzed electron transport in PSI and PSII. Light–response curves provide valuable insight into photosystem efficiency under increasing irradiance and the allocation of photosynthetically active radiation (PAR). Absorbed light energy can follow three principal fates: photochemical conversion [Y(I) or Y(II)], regulated energy dissipation as heat via NPQ [Y(NPQ)], or non-regulated energy loss as heat/fluorescence [Y(NO)] (Klughammer and Schreiber, 1994). Immediately upon illumination, Y(II) decreased from ∼0.8 to 0.6–0.5 in Col-0, *npq4-1*, and oePsbS, whereas in the double and triple oePsbSoeβCA lines it dropped more sharply to 0.4–0.3 (Fig. 3B). Above a PAR of 300 µmol m^–2^ s^–1^, differences among genotypes largely disappeared. The reduced Y(II) in oePsbSoeβCA lines was accompanied by elevated Y(NPQ) (Fig. 3D), indicating stronger regulated heat dissipation. Y(NO) was similar across genotypes at low PAR (Fig. 3F), but diverged at higher irradiance, with oePsbS showing reduced and *npq4-1* increased Y(NO). Variations in ΔpH-dependent Y(NPQ) largely determined the overall Y(II) patterns. The photochemical yield of PSI, Y(I), was higher in double and triple oePsbSoeβCA lines compared with other genotypes (Fig. 3A). At irradiances >58 µmol m^–2^ s^–1^, these lines exhibited increased donor-side limitation of PSI [Y(ND)] (Fig. 3C), accompanied by decreased acceptor-side limitation [Y(NA)] (Fig. 3E). Together, these data indicate that *βCA1* and *βCA2* overexpression in a single PsbS-overexpressing background disrupts PSI:PSII stoichiometry, shifting electron transport toward PSI. This is consistent with enhanced CET, which serves to protect both donor and acceptor sides of PSI, and may also reflect retrograde signaling from chloroplasts (Foyer *et al.*, 2012).

### *βCA1* and *βCA2* overexpression in the oePsbS background induces a molecular response to biotic and abiotic stresses after bicarbonate treatment

**Fig. 4.**
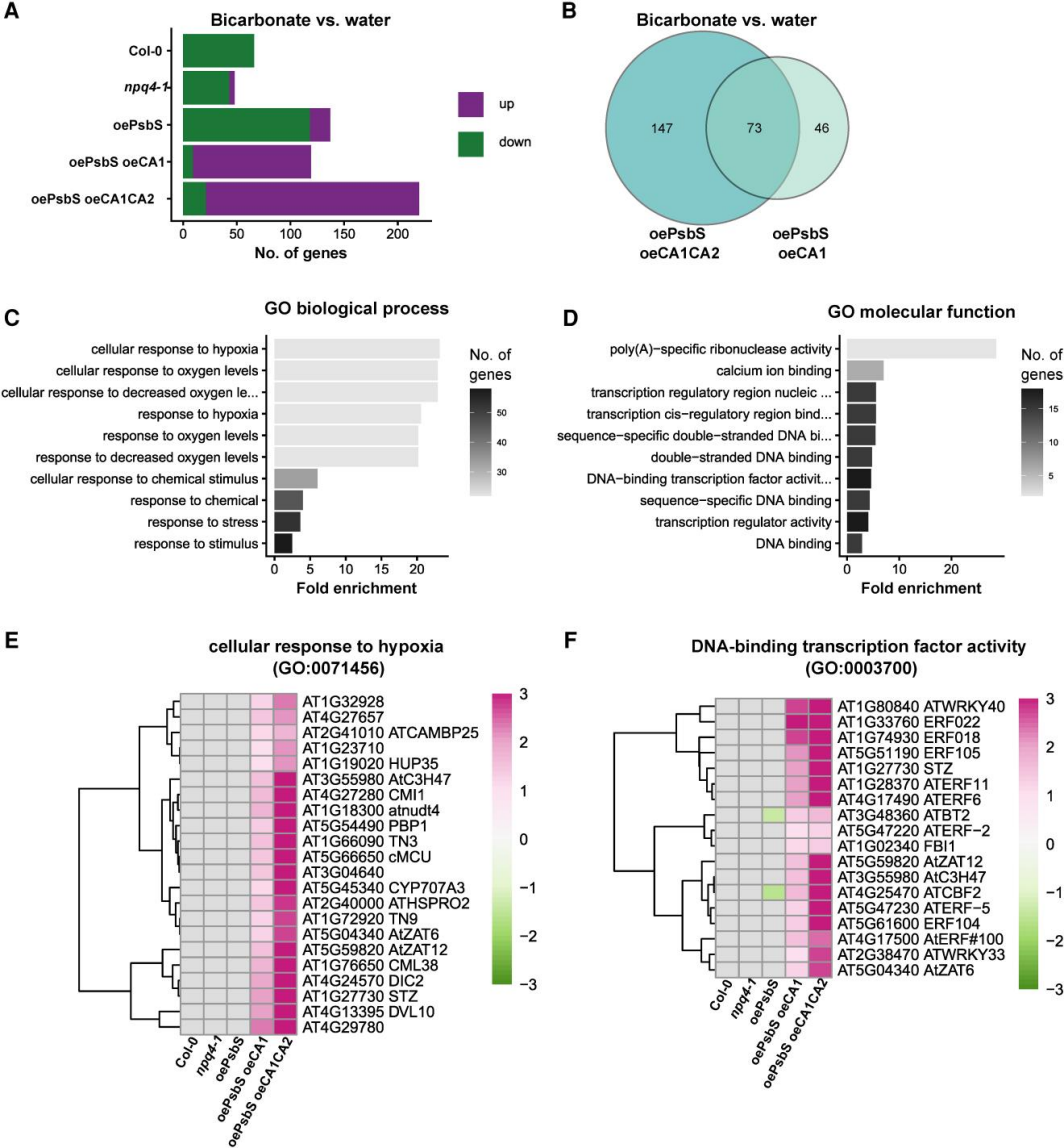
Differential gene expression pattern after bicarbonate versus water treatment of Col-0, *npq4-1*, oePsbS, double (oePsbSoeβCA1), and triple (oePsbSoeβCAβCA2) overexpressing transgenic *A. thaliana* lines cultivated in ambient laboratory conditions. (A) The diagram presents the number of up- and down-regulated genes conversely regulated in analyzed genotypes after bicarbonate versus water treatment. (B) The Venn diagram presents pools of genes regulated by bicarbonate in the oePsbSoeβCA1 and oePsbSoeβCAβCA2 plants. A total of 147 and 46 genes, respectively, were differentially regulated in these genotypes, and 73 genes were in common. (C and D) Gene Ontology (GO) analysis of oePsbSoeβCA1- and oePsbSoeβCAβCA2-specific genes that are affected by bicarbonate treatment (in total, 73 genes were analyzed). (E and F) Clustering of genes specific to oePsbSoeβCA1 and oePsbSoeβCAβCA2 plants belonging to ‘cellular response to hypoxia’ and ‘DNA-binding transcription factor activity’ GO groups.

Double and triple oePsbSoeβCA lines differed from Col-0, *npq4-1*, and oePsbS not only physiologically but also at the transcriptional level. To assess changes in gene expression, RNA-seq was performed on 4-week-old plants under control conditions and supplied with 3 mM HCO_3_^−^. Differentially expressed genes (DEGs) were identified for each genotype independently (Fig. 4). In Col-0, *npq4-1*, and oePsbS, bicarbonate fertilization predominantly down-regulated stress-related genes and transcription factors (TFs). In contrast, the majority of DEGs in oePsbSoeβCA lines were up-regulated (Fig. 4A). Among 266 deregulated genes in the double and triple lines, 73 were shared between both genotypes (Fig. 4B). GO analysis revealed two major functional categories: genes involved in cellular response to hypoxia (>20 of 186 annotated in Arabidopsis) and TFs regulating responses to drought, freezing, HL, salinity, cold, and pathogens (Fig. 4C–F). Notably, HCO_3_^−^ treatment suppressed two such TFs in oePsbS but not in oePsbSoeβCA lines, suggesting that elevated βCA activity counteracted this repression (Fig. 4F). These included CBF2, a DREB/AP2 factor central to abiotic stress tolerance, and BT2, a component of the TAC1-mediated telomerase activation pathway. Furthermore, five DREB-regulated stress-responsive genes (WRKY40, WRKY33, ZAT6, ZAT12, and ERF105) were among the 266 DEGs specifically induced in the βCA overexpression lines following bicarbonate fertilization (Fig. 4E, F).

## Discussion

Plants often absorb more light energy than is required for photosynthesis, and the PsbS protein plays a central role in dissipating excess excitation energy through NPQ. Optimizing the partitioning of absorbed light between photochemistry, heat dissipation, and fluorescence is therefore critical for improving biomass production and crop yield. Based on current knowledge and our previous findings, we hypothesized that overexpression of *βCA1* and *βCA2* in a single PsbS-overexpressing background would improve HCO_3_^−^ utilization and enhance photosynthetic efficiency under fluctuating environmental conditions.

Plants can acquire carbon both from the atmosphere and from water, where HCO_3_^−^ may enter roots, be transported through the xylem vessels to leaves, and be converted to CO_2_ by CAs before fixation (Rao and Wu, 2017; Poschenrieder *et al.*, 2018). In our study, bicarbonate fertilization stimulated biomass accumulation, but this effect was strongly light dependent. Under controlled laboratory conditions, all genotypes produced slightly less biomass than water-treated controls, whereas under greenhouse conditions, double and triple oePsbSoeβCA lines accumulated significantly more fresh and dry mass than oePsbS and *npq4-1* (Supplementary Fig. S4). These results suggest that βCAs enhance inorganic carbon use efficiency, particularly under fluctuating light, pointing to a potential additive role of βCA1 and βCA2 or a predominant contribution of βCA2. This interpretation is consistent with previous reports showing that moderate bicarbonate supply can stimulate growth when sufficient ATP and NADPH are available (Dąbrowska-Bronk *et al.*, 2016; Wang *et al.*, 2016; Fantuzzi *et al.*, 2023). It also aligns with findings that HCO_3_^−^ may directly regulate PSII activity and the assembly of the oxygen-evolving complex (Shitov *et al.*, 2018; Shevela *et al.*, 2020).

The efficiency of carbon assimilation depends on the balance between Δψ and ΔpH within the pmf (Mitchell, 1961; Kramer *et al*., 2003; Górecka *et al*., 2020). We observed that oePsbSoeβCA lines maintained higher ΔpH and slightly enhanced NPQ compared with oePsbS (Fig. 1D, F), thereby improving CO_2_/HCO_3_^−^ availability for photochemistry. This effect contributed to greater biomass accumulation than in oePsbS and *npq4-1*, although not exceeding that of Col-0 (Fig. 1A, B). These results are consistent with the protective role of ΔpH in preventing over-reduction of PSI and PSII (Suorsa *et al*., 2013) and with the well-established function of NPQ in limiting photooxidative damage (Logan *et al*., 2008; Ruban, 2016). In contrast, increased Δψ has been associated with singlet oxygen production and photodamage (Davis *et al*., 2017). Thus, elevated activity of βCAs may help shift pmf partitioning toward ΔpH, supporting more effective photoprotection under fluctuating light conditions.

The role of CAs in photoprotection has been previously proposed. Arabidopsis *βca* mutants exhibited reduced ion leakage and enhanced NPQ after HCO_3_^−^ treatment (Dąbrowska-Bronk *et al*., 2016), while HCO_3_^−^ fertilization increased carotenoids and xanthophyll pigments known to protect PSII. Similarly, *αca4* mutants displayed reduced Lhcb proteins but increased PsbS abundance and VAZ cycle activity (Rudenko *et al*., 2018, 2020). In our study, overexpression of *βCA1* or *βCA1βCA2* in the oePsbS background reduced PsbS (Supplementary Fig. S1), D1, Lhcb1, and Lhcb2 levels (Supplementary Fig. S5), and surprisingly improved tolerance to HL stress (Fig. 2B). These plants also exhibited a higher Chl *a*/*b* ratio (Supplementary Fig. S6A) but unchanged carotenoid levels (Supplementary Fig. S6B), suggesting that βCAs modulate antenna composition to optimize absorbed energy fate and distribution.

Under DCMU treatment, which blocks PSII electron transfer and increases susceptibility to oxidative stress (Gawroński *et al*., 2021), oePsbSoeβCA lines maintained higher QY_max_ than other genotypes (Fig. 2A), indicating reduced inhibition of linear electron flow. However, under control conditions, PSII electron transport efficiency, donor-side limitation, and pmf partitioning were impaired in these lines (Supplementary Fig. S7; Fig. 3C, and Fig. 1E, respectively), consistent with partial photoinhibition and an altered ATP/NADPH balance. In response to these imbalances, alternative CET pathways were probably activated, involving ferredoxin-dependent PQ reduction and NPQ activation (Foyer *et al*., 2012). This interpretation is supported by previous work showing that CET is essential for protecting PSI under fluctuating light (Kono *et al*., 2014; Walker *et al*., 2014), and is impaired in oePsbS plants, making them more sensitive to rapid changes in irradiance. Our greenhouse experiments are consistent with this compensatory mechanism: double and triple oePsbSoeβCA lines exhibited enhanced growth under variable light compared with oePsbS, reflecting the contribution of βCAs to stabilizing electron transport and photoprotection under natural fluctuations (Supplementary Fig. S4).

Gas exchange analyses provided additional evidence for the physiological effects of *βCA1* and *βCA2* overexpression. Double and triple oePsbSoeβCAs line exhibited reduced stomatal conductance and transpiration, resulting in improved WUE, while CO_2_ assimilation remained stable across genotypes (Supplementary Fig. S3). These findings align with previous studies demonstrating that Arabidopsis βCAs regulate stomatal closure via ABA-independent CO_2_ signaling, enhancing WUE without compromising carbon fixation (DiMario *et al*., 2017; Kolbe *et al*., 2018). The lower biomass of oePsbSoeβCA lines compared with Col-0 under ambient conditions is therefore likely to be linked to stomatal limitations. However, these genotypes may gain a physiological advantage under elevated CO_2_ and higher temperatures, as suggested previously (DiMario *et al*., 2016; Zarrin Ghalami *et al*., 2026).

At the transcriptome level, HCO_3_^−^ treatment revealed contrasting stress responses among the genotypes. Col-0, *npq4-1*, and oePsbS plants showed broad down-regulation of stress-responsive genes, whereas oePsbSoeβCA lines strongly induced hypoxia- and reactive oxygen species (ROS)-related TFs, including ZAT6, ZAT12, WRKY33, WRKY40, and ERF105 (Fig. 4). These regulators are known to coordinate ROS detoxification and enhance tolerance to multiple abiotic stresses (Hua *et al*., 2001; Davletova *et al*., 2005; Chen *et al*., 2016; Bolt *et al*., 2017; Szechyńska-Hebda *et al*., 2022; Kamran *et al*., 2025). Hormone-associated signaling pathways, including ethylene, JA, and SA, also contribute to this crosstalk (Mateo *et al*., 2006; Gururani *et al*., 2015; De Ollas *et al.*, 2021; Pan *et al*., 2022; Molinari *et al.,* 2023; Kamran *et al*., 2025, 2026; Zarrin Ghalami *et al*., 2026). Enhanced expression of ZAT6 and ZAT12 in oePsbSoeβCA lines is particularly noteworthy, as they promote ROS scavenging and anthocyanin synthesis during oxidative stress (Rizhsky *et al*., 2004; Shi *et al*., 2018). Whether this transcriptional activation reflects hypersensitivity to moderate HCO_3_^−^ stress or confers a broader stress tolerance remains unresolved. In Arabidopsis, moderate concentrations of HCO_3_^−^ (3–5 mM) stimulate plant growth, enhance NPQ, and increase the expression of C_3_ photosynthetic enzymes (Dąbrowska-Bronk *et al.*, 2016). In this context, the strong transcriptional activation observed exclusively in the βCA-overexpressing lines following 3 mM HCO_3_^−^ fertilization is unlikely to reflect a genuine physiological stress response. Instead, our physiological, molecular, and phenotypic analyses indicate that this induction represents a heightened responsiveness to environmental stimuli that do not elicit stress responses in genotypes with endogenous CA levels. Given that CAs modulate CO_2_/HCO_3_^−^ interconversion and contribute to ΔpH regulation, elevated βCA activity may alter chloroplast homeostasis in a way that lowers the activation threshold for redox- and ROS-dependent transcriptional networks. Collectively, these findings confirm that CAs play an important role in plant responses to both biotic and abiotic stresses, although their effects can be contrasting and depend strongly on the specific stress factor, its intensity, and the plant species involved (Polishchuk, 2021).

Balancing carbon assimilation with ΔpH-dependent photoprotection is fundamental for plant performance under fluctuating environments. While βCAs and PsbS are both central regulators of these processes, their functional relationship has not been previously reported. The key and unexpected outcome of our work was the consistent reduction of the PsbS protein level in *βCA-*overexpressing lines. Double and triple transgenic lines showed decreased PsbS abundance in both the oePsbS (Supplementary Fig. S1C, D) and Col-0 backgrounds (Supplementary Fig. S2A, C), whereas β*ca* mutants accumulated higher levels of PsbS (Supplementary Fig. S2B, D). This inverse correlation suggests the existence of a feedback mechanism linking CA activity to the regulation of PsbS protein, most probably through the modulation of ΔpH and the maintenance of a balanced pmf. These findings uncover a new layer of chloroplast retrograde control linking HCO_3_^−^ metabolism to photoprotection, expanding the conceptual framework of how plants balance carbon assimilation with energy dissipation.

In conclusion, overexpression of Arabidopsis *βCA1* and *βCA2* in the oePsbS background altered the balance between carbon utilization, photoprotection, WUE, and transcriptional stress responses. Although these plants exhibited enhanced WUE and NPQ, they also activated compensatory photoprotective and stress response pathways, ultimately resulting in lower biomass relative to Col-0. The strong light dependency of bicarbonate fertilization and the apparent ΔpH-linked feedback between βCAs and PsbS highlight the complexity of this regulatory network. Future work employing Arabidopsis Col-0oeβCAs and *βcas*/*npq4-1* crossing lines will be crucial to better understand these mechanisms and to determine whether overexpression of βCAs improves stress tolerance or simply shifts the threshold for activation of stress-responsive transcriptional networks.

## Supplementary Material

erag155_Supplementary_Data

## Data Availability

The RNA-seq data underlying this article are available in the Gene Expression Omnibus (GEO) Database/NCBI, and can be accessed with the accession number GSE325023. The remaining data that support the findings of this study are available from the corresponding author upon request.

## Supplementary data

The following supplementary data are available at *JXB* online.

Fig. S1. Analysis of Col-0, *npq4-1*, oePsbS, double (oePsbSoeβCA1), and triple (oePsbSoeβCA1βCA2) transgenic plants.

Fig. S2. Analysis of Col-0, double (Col-0oeβCA1), triple (Col-0oeβCA1βCA2) transgenic plants and β*cas* mutants.

Fig. S3. Gas exchange analysis of Col-0, *npq4-1*, oePsbS, double (oePsbSoeβCA1), and triple (oePsbSoeβCA1βCA2) transgenic plants.

Fig. S4. Biomass production under variable light conditions in Col-0, *npq4-1*, oePsbS, double (oePsbSoeβCA1), and triple (oePsbSoeβCA1βCA2) transgenic plants.

Fig. S5. Light-harvesting complex II analysis of Col-0, *npq4-1*, oePsbS, double (oePsbSoeβCA1), and triple (oePsbSoeβCA1βCA2) transgenic plants.

Fig. S6. Antioxidant content in Col-0, *npq4-1*, oePsbS, double (oePsbSoeβCA1), and triple (oePsbSoeβCA1βCA2) transgenic plants.

Fig. S7. PSII and PSI electron transport rate in Col-0, *npq4-1*, oePsbS, double (oePsbSoeβCA1), and triple (oePsbSoeβCA1βCA2) transgenic plants.

Table S1. List of primer sequences used in this study.

Table S2. Dilution of primary and secondary antibodies used in western blot analysis.

## Abbreviations

CA: carbonic anhydrase
CET: cyclic electron transport
HCO_3_^−^: bicarbonate
NPQ (qE): non-photochemical quenching
