# Supplementary material for: Crosstalk between β-carbonic anhydrases and PsbS in the regulation of photosynthesis and stress tolerance in Arabidopsis
Source: J Exp Bot. 2026 Apr 2;77(14):4607–19. doi: 10.1093/jxb/erag155 (PMC13415952; doi:10.1093/jxb/erag155)
Supplement: erag155_Supplementary_Data [file erag155_supplementary_data.pdf]

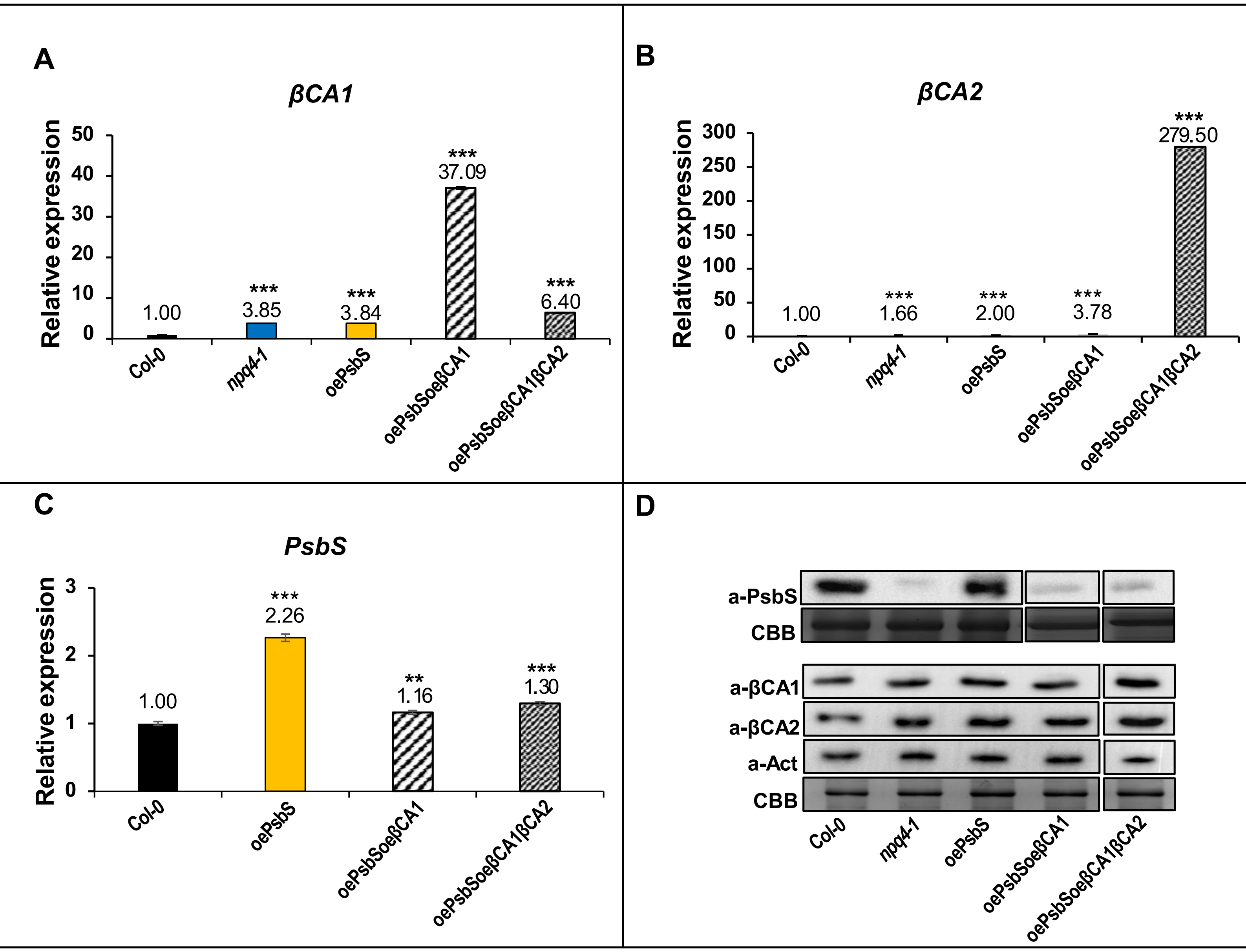

Supplementary Figure S1. Analysis of Col-0, *npq4-1*, oePsbS, double (oePsbSoeβCA1), triple (oePsbSoeβCA1βCA2) transgenic plants. Expression levels of *βCA1* **(A)**, *βCA2* **(B)** and *PsbS* **(C,D)** genes in leaves of four-week-old Arabidopsis plants **(A-C)** cultivated in a growing chamber under normal light conditions 120 µE. Transcripts levels were normalized to *PP2AA3* expression. Data are shown as a mean ± SD of three biological samples (n=9). Asterisks indicate a statistically significant difference compared with Col-0 plants as revealed by one-way ANOVA; α=0,05; <0,05*; <0,01**; <0,001***. **(D)** Western blot analysis of PsbS, βCA1 and βCA2 proteins in leaves of four-week-old Arabidopsis plants cultivated in a growing chamber under normal light conditions 120 µE. Results for oePsbSoeβCA1 and oePsbSoeβCA1βCA2 are shown as an overage value of three independent transgenic lines **(A-D)**.

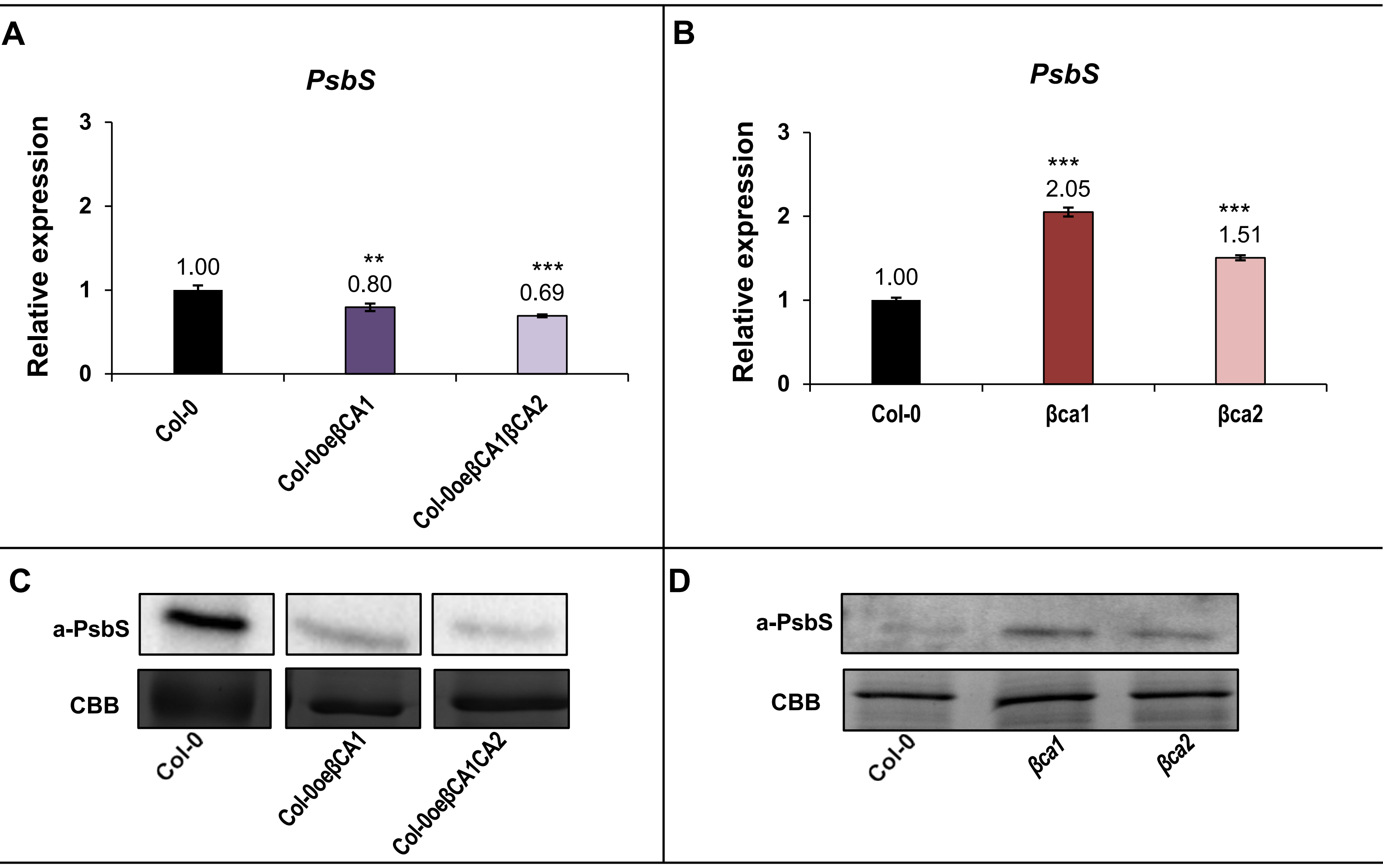

Supplementary Figure S2. Analysis of Col-0, double (Col-0oeβCA1), triple (Col-0oeβCA1βCA2) transgenic plants and β*cas* mutants. Expression levels of *PsbS* genes in leaves of four-week-old Arabidopsis plants **(A)** and Arabidopsis β*cas* mutants **(B)** cultivated in a growing chamber under normal light conditions 120 μE. Transcripts levels were normalized to *PP2AA3* expression. Data are shown as a mean ± SD of three biological samples (n=9). Asterisks indicate a statistically significant difference compared with Col-0 plants as revealed by one-way ANOVA; α=0,05; <0,05*; <0,01**; <0,001***. Western blot analysis of PsbS protein in leaves of four-week-old Arabidopsis plants **(C)** and β*cas* mutants **(D)** cultivated in a growing chamber under normal light conditions 120 μE. Results are shown as an overage value of three independent transgenic lines.

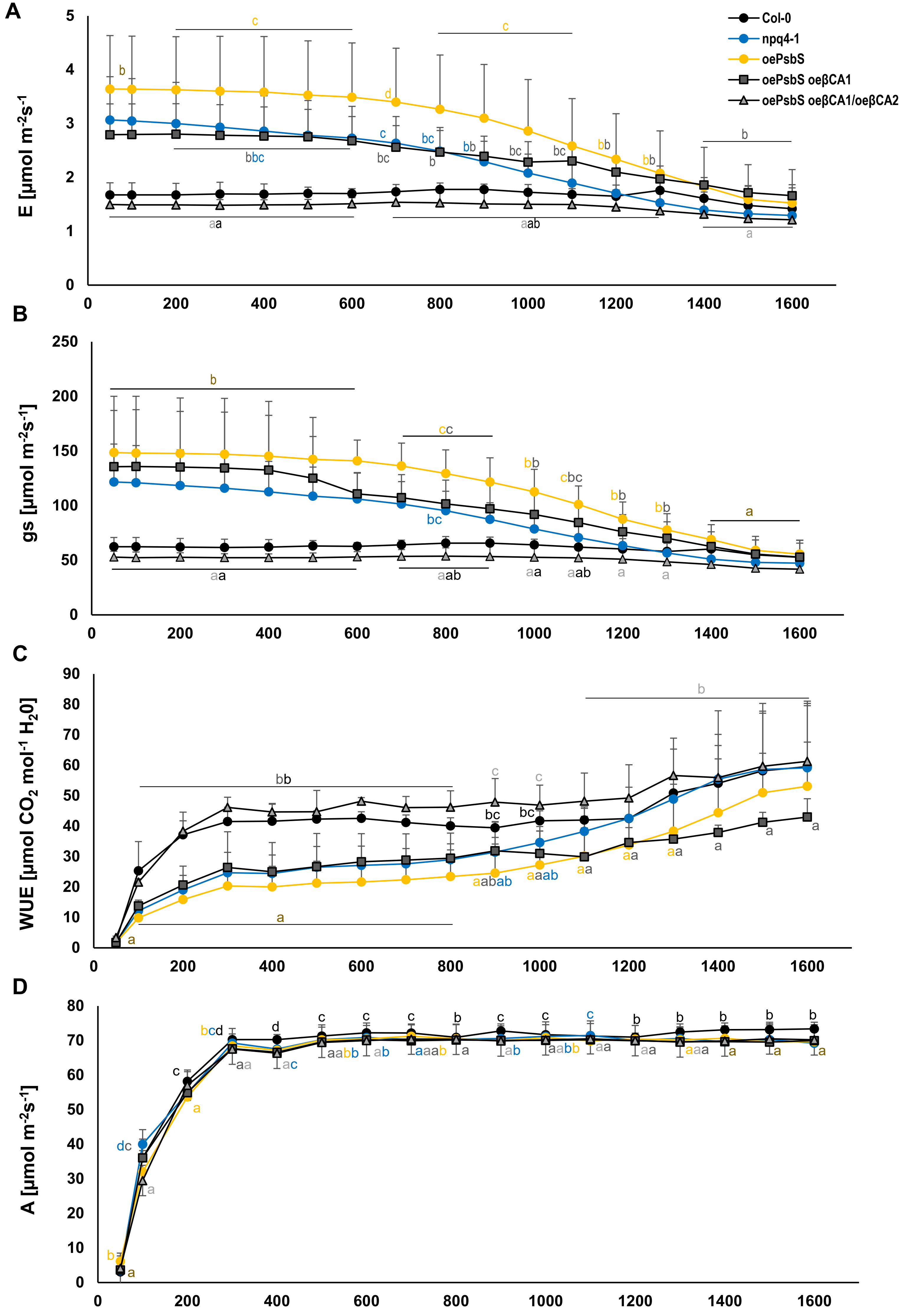
A
B
C
D
Col-0
npq4-1
oePsbS
oePsbS oeβCA1
oePsbS oeβCA1/oeβCA2
E [μmol m-2s-1]
gs [μmol m-2s-1]
WUE [μmol CO2 mol-1 H20]
A [μmol m-2s-1]
0
200
400
600
800
1000
1200
1400
1600

Supplementary Figure S3. Gas exchange analysis of Col-0, *npq4-1*, oePsbS, double (oePsbSoeβCA1) and triple (oePsbSoeβCA1βCA2) transgenic plants (A-D). (A) E-transpiration rate, **(B)** gs-stomatal conductance, **(C)** WUE-water use efficiency, and **(D)** A-assimilation parameters in four-week-old Arabidopsis plants cultivated in a growing chamber under normal light conditions 120 µE. One-way ANOVA Fisher's least significant difference (LSD) test, 95% c.I, being used to estimate the difference between each pair of means. Data are shown as a mean of three biological samples ± SD (n=6). Results for oePsbSoeβCA1 and oePsbSoeβCA1βCA2 are shown as an overage value of three independent transgenic lines.

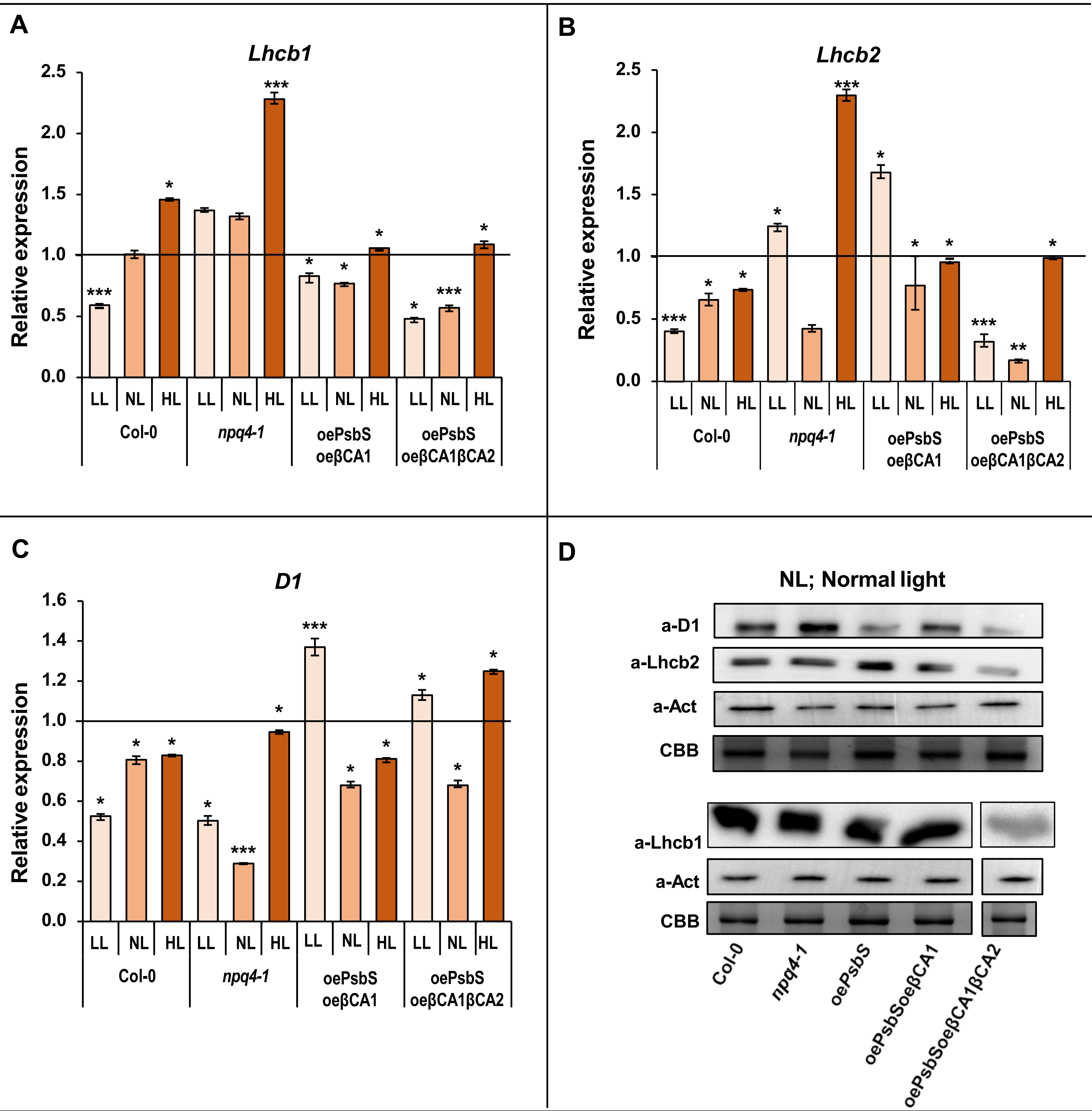

Supplementary Figure S4. Light-Harvesting Complex II (LHC II) analysis of Col-0, *npq4-1*, oePsbS, double (oePsbSoeβCA1) and triple (oePsbSoeβCA1βCA2) transgenic plants. Expression of *Lhcb1* **(A)**, *Lhcb2* **(B)** and *D1* **(C)** genes in leaves of four-week-old Arabidopsis plants cultivated in a growing chamber under variable light conditions: Low light (LL) - 80; Normal light (NL) -120; High light (HL) - 800 µE. Transcripts levels were normalized to *PP2AA3* expression. Data are shown as a mean ± SD of three biological samples (n=9). Asterisks indicate a statistically significant difference compared with oePsbS plants (oePsbS=1) as revealed by one-way ANOVA; α=0,05; <0,05*; <0,01**; <0,001***. **(D)** Western blot analysis of D1, Lhcb1, Lhcb2 proteins in leaves of four-week-old Arabidopsis plants cultivated in a growing chamber under normal light conditions 120 µE. Results for oePsbSoeβCA1 and oePsbSoeβCA1βCA2 are shown as an overage value of three independent transgenic lines.

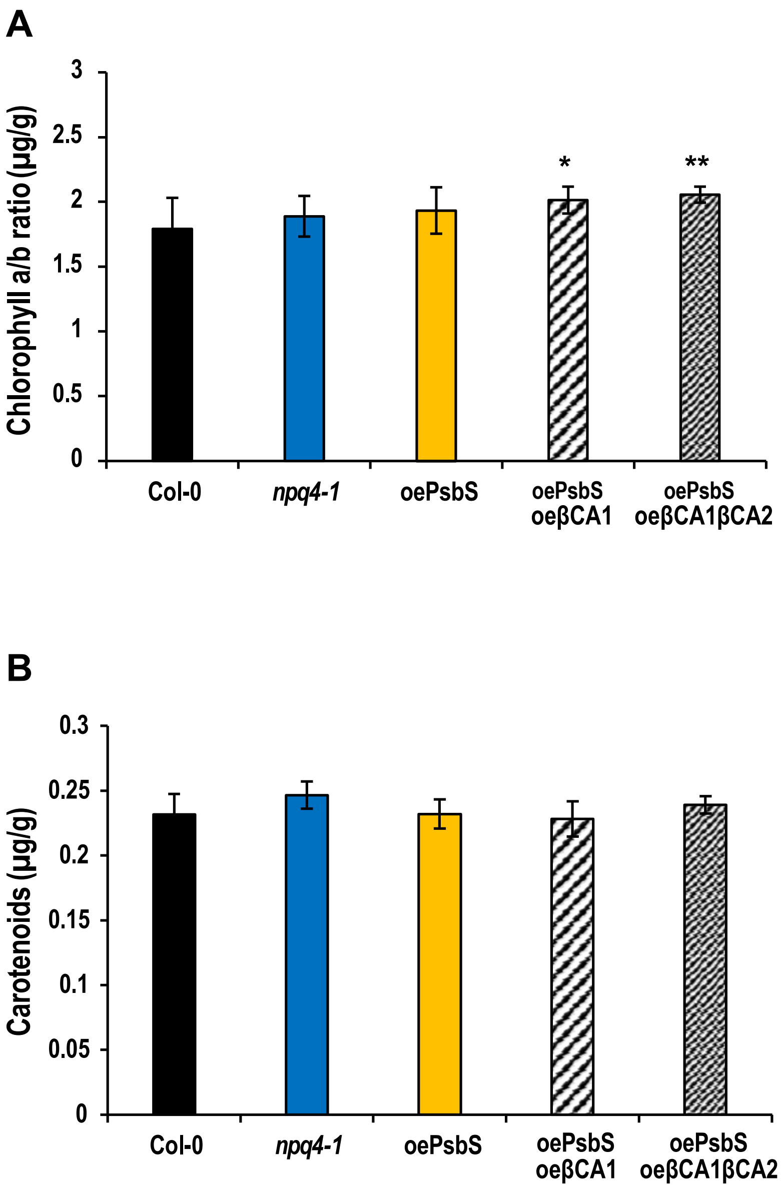

Supplementary Figure S5. Antioxidant content in Col-0, *npq4-1*, oePsbS, double (oePsbSoeβCA1) and triple (oePsbSoeβCA1βCA2) transgenic plants. Chlorophyll a/b ratio **(A)** and carotenoids content **(B)** were measured in four-week-old Arabidopsis plants cultivated in a growing chamber under normal light conditions 120 µE. Data are shown as a mean ± SD of three biological samples (n=9). Asterisks indicate a statistically significant difference compared with Col-0 plants as revealed by one-way ANOVA; α=0,05; <0,05*; <0,01**; <0,001***. Results for oePsbSoeβCA1 and oePsbSoeβCA1βCA2 are shown as an overage value of three independent transgenic lines.

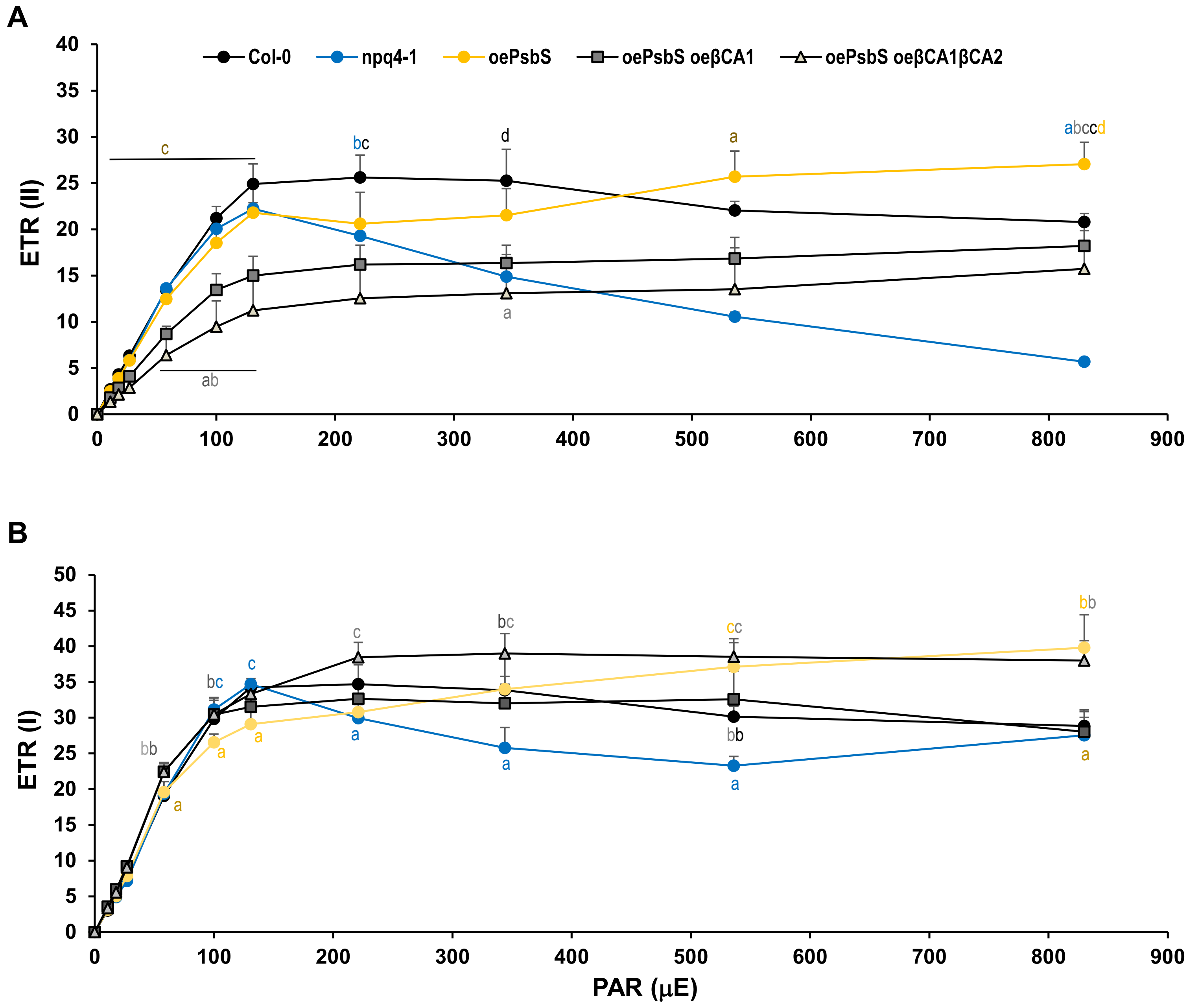

Supplementary Figure S6. Photosystem II and I electron transport rate (ETR) in Col-0, *npq4-1*, oePsbS, double (oePsbSoeβCA1) and triple (oePsbSoeβCA1βCA2) transgenic plants. (A) Photosystem II and **(B)** Photosystem I **E**ectron **T**ransport **R**ate measured in four-week-old Arabidopsis plants cultivated in a growing chamber under normal light conditions 120 µE. One-way ANOVA Fisher's least significant difference (LSD) test, 95% c.l, being used to estimate the difference between each pair of means. Data are shown as a mean ± SD of three biological samples (n=6). Results for oePsbSoeβCA1 and oePsbSoeβCA1βCA2 are shown as an overage value of three independent transgenic lines.

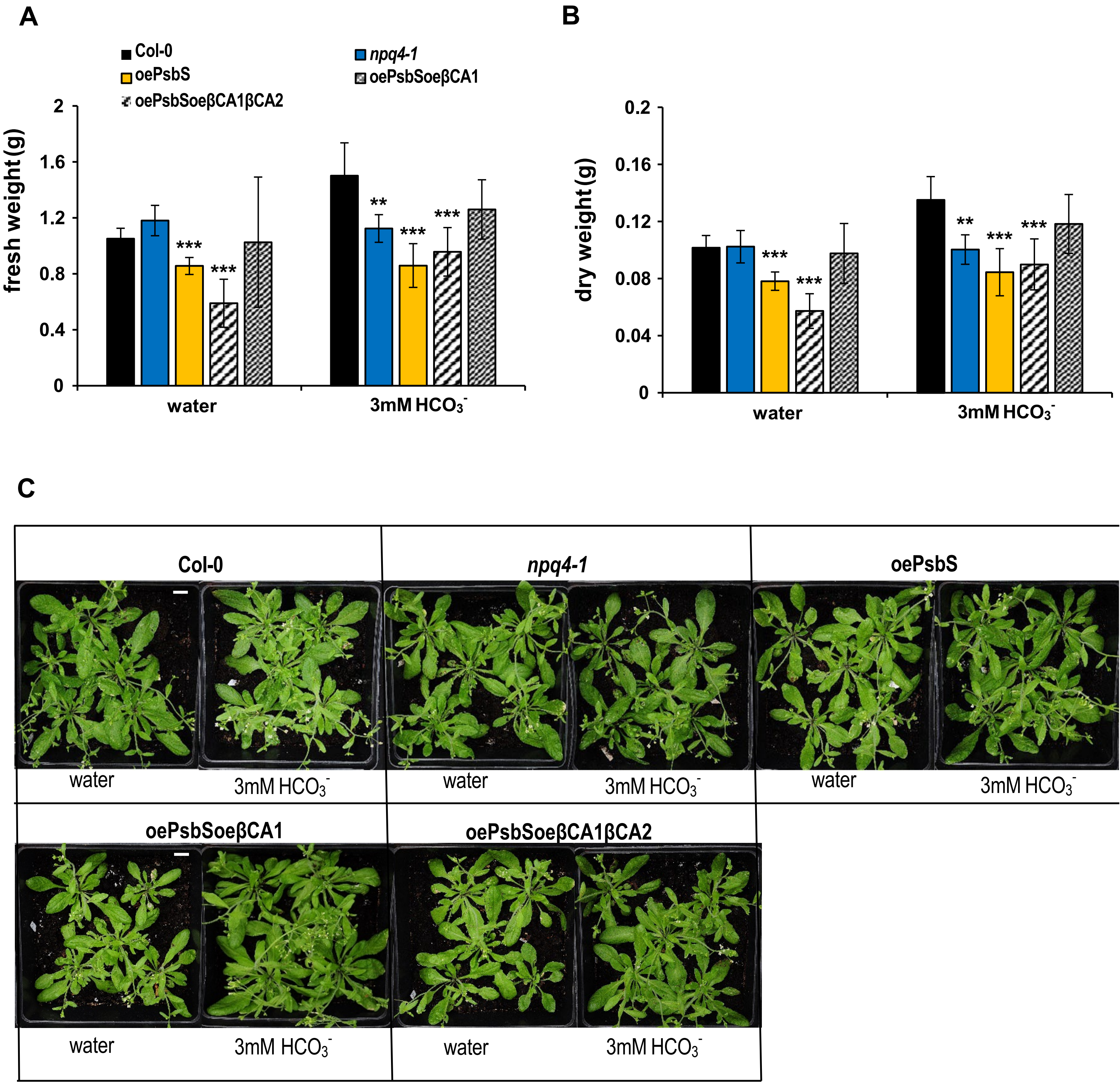

Supplementary Figure S7. Biomass production under variable light conditions in Col-0, *npq4-1*, oePsbS, double (oePsbSoeβCA1) and triple (oePsbSoeβCA1βCA2) transgenic plants. (A) Fresh and **(B)** dry weight measured in four-week-old Arabidopsis plants cultivated under greenhouse conditions, fertilized with water or 3mM bicarbonate. Data are shown as a mean ± SD of three biological samples (n=10). Asterisks indicate a statistically significant difference compared with Col-0 plants as revealed by one-way ANOVA; α=0,05; <0,05*; <0,01**; <0,001***. Results for oePsbSoeβCA1 and oePsbSoeβCA1βCA2 are shown as an overage value of three independent transgenic lines. **(C)** Morphology of four-week-old Arabidopsis plants grown in a greenhouse condition, fertilized with water or 3mM bicarbonate, scale bar=1cm.

Supplementary Table S1. List of primer sequences used in thisstudy

RT-qPCR primers	Forward	Reverse
PP2AA3 (AT1G13320)	5'-TAACGTGGCCAAAATGATGC-3'	5'-GTTCTCCACAACCGCTTGGT-3'
βCA1 (AT3G01500)	5'-TCTTCCACCGTCGCTTGCCTC-3'	5'-CTCGTTACGGATAAGCGTTGG-3'
βCA2 (AT5G14740)	5'-GTCCCTCTCATCGCCTGTCT-3'	5'-CTTCTCAGTTCAAGTTTGAAG-3'
PsbS (AT1G44575)	5'-AGGAAGATTGGCACAGTTGG-3'	5'-ATGGCAGCGAAGAAGAAGAA-3'
Lhcb1 (AT1G29920)	5'-CCTTCGCTACCAACTTCGTC-3'	5'-GAGTAACAAACCGGATACA-3'
Lhcb2 (AT2G05070)	5'-TCTCGGAAAACACACCATCA-3'	5'-TGCCCATCTACTGTGGATCA-3'
D1 (*PsbA;* ATCG00020)	5'-GCTATACAACGGCGGTCCTTA-3'	5'-GTTCCCACTCACGACCCATAT-3'

Supplementary Table S2. Dilution of primary and secondary antibodies used in Western blot analysis.

Primary antibody	Dilution	Catalog number	Manufacturer
anti-PsbS	1:10000	AS09 533	Agrisera, Sweden
anti-βCA1	1:20000	AS19 4321	Agrisera, Sweden
anti-βCA2	1:10000	PHY1628S	PhytoAB, USA
anti-D1	1:10000	AS11 1786	Agrisera, Sweden
anti-Lhcb1	1:5000	AS09 522	Agrisera, Sweden
anti-Lhcb2	1:5000	AS01 003	Agrisera, Sweden
anti-Actin	1:10000	SAB4301137	Merck, Germany
**Secondary antibody**	**Dilution**	**Catalog number**	**Manufacturer**
anti-rabbit HRP	1:10000	# 31460	Thermo Fisher Scientific, USA
